# Results of the first recorded evaluation of a national gestational diabetes mellitus register: Challenges in screening, registration, and follow-up for diabetes risk

**DOI:** 10.1371/journal.pone.0200832

**Published:** 2018-08-08

**Authors:** Douglas I. R. Boyle, Vincent L. Versace, James A. Dunbar, Wendy Scheil, Edward Janus, Jeremy J. N. Oats, Timothy Skinner, Sophy Shih, Sharleen O’Reilly, Ken Sikaris, Liza Kelsall, Paddy A. Phillips, James D. Best, on behalf of MAGDA Study Group

**Affiliations:** 1 Department of General Practice, Faculty of Medicine, Dentistry & Health Sciences, University of Melbourne, Melbourne, Victoria, Australia; 2 School of Medicine, Deakin Rural Health, Deakin University, Warrnambool, Victoria, Australia; 3 Public Health & Clinical Systems, SA Health, Adelaide, South Australia, Australia; 4 Discipline of Obstetrics & Gynaecology, The University of Adelaide, Adelaide, South Australia, Australia; 5 Department of Medicine-Western Health, Melbourne Medical School, The University of Melbourne, Melbourne, Victoria, Australia; 6 General Internal Medicine Unit, Western Health, St Albans, Victoria, Australia; 7 Melbourne School of Population and Global Health, University of Melbourne, Melbourne, Victoria, Australia; 8 School of Psychological and Clinical Sciences, Charles Darwin University, Casuarina, Northern Territory, Australia; 9 Centre for Population Health Research, Faculty of Health, Deakin University, Melbourne, Australia; 10 School of Agriculture and Food Science, University College, Dublin, Ireland; 11 Melbourne Pathology, Collingwood, Victoria, Australia; 12 Health Intelligence Unit, System Intelligence & Analytics, Department of Health and Human Services, Melbourne, Victoria, Australia; 13 SA Health, Adelaide, South Australia, Australia; 14 Lee Kong Chian School of Medicine, Nanyang Technological University, Singapore, Singapore; Florida International University Herbert Wertheim College of Medicine, UNITED STATES

## Abstract

**Objective:**

Gestational Diabetes Mellitus (GDM) increases the risk of type 2 diabetes. A register can be used to follow-up high risk women for early intervention to prevent progression to type 2 diabetes. We evaluate the performance of the world’s first national gestational diabetes register.

**Research design and methods:**

Observational study that used data linkage to merge: (1) pathology data from the Australian states of Victoria (VIC) and South Australia (SA); (2) birth records from the Consultative Council on Obstetric and Paediatric Mortality and Morbidity (CCOPMM, VIC) and the South Australian Perinatal Statistics Collection (SAPSC, SA); (3) GDM and type 2 diabetes register data from the National Gestational Diabetes Register (NGDR). All pregnancies registered on CCOPMM and SAPSC for 2012 and 2013 were included–other data back to 2008 were used to support the analyses. Rates of screening for GDM, rates of registration on the NGDR, and rates of follow-up laboratory screening for type 2 diabetes are reported.

**Results:**

Estimated GDM screening rates were 86% in SA and 97% in VIC. Rates of registration on the NGDR ranged from 73% in SA (2013) to 91% in VIC (2013). During the study period rates of screening at six weeks postpartum ranged from 43% in SA (2012) to 58% in VIC (2013). There was little evidence of recall letters resulting in screening 12 months follow-up.

**Conclusions:**

GDM Screening and NGDR registration was effective in Australia. Recall by mail-out to young mothers and their GP’s for type 2 diabetes follow-up testing proved ineffective.

## Introduction

Women with previous GDM have a sevenfold higher risk of developing type 2 diabetes mellitus than women who have not had GDM [1]. Recall registers have a well-recognised role for managing populations at high risk. Australia has had two registries for gestational diabetes mellitus (GDM) that have been created to support the follow-up of mothers who have had GDM to help prevent progression to type 2 diabetes or identify it at an early stage. There was a previous state-based South Australia Gestational Diabetes Recall Register (2002-June 2011) and a current National Gestational Diabetes Register (NGDR, from July 2011). The NGDR is managed by the National Diabetes Support Scheme (NDSS) under the auspices of Diabetes Australia. Women meeting the criteria for GDM are registered with NGDR by diabetes educators. These registers support follow-up of women for regular diabetes screening and lifestyle modification to reduce their risk of developing type 2 diabetes through periodic letters to both the patient and the GP. Registration on the GDM registers is not mandated. In addition to the GDM registers, each state maintains a Birth Register with mandated reporting of all births and includes recording GDM.

Lifestyle modification can reduce type 2 diabetes risk by 58% [2,3]. The NGDR provides information and regular screening reminder. It is administered by Diabetes Australia, an NGO, on behalf of the Australian Government. Women are registered with NGDR following diagnosis of GDM at the 26–28 week Oral Glucose Tolerance Test (OGTT) which entitles them to subsidised medication and equipment for managing diabetes during their pregnancy.

Our study sought to evaluate for the first time the extent to which a National Gestational Diabetes Register contributes to prevention of subsequent diabetes among women diagnosed with GDM. Specifically this study used data linkage to evaluate:

1. The completeness of state-based GDM screening during pregnancy
2. The completeness of registration of mothers with GDM on the NGDR
3. The impact of register letters on mothers and GPs to influence mothers to have follow-up blood tests for diabetes risk as indicated through pathology test results.

The study linked information from the NGDR, perinatal data collections from two states, and blood test results from pathology laboratories in both states.

## Materials and methods

**Fig 1:**
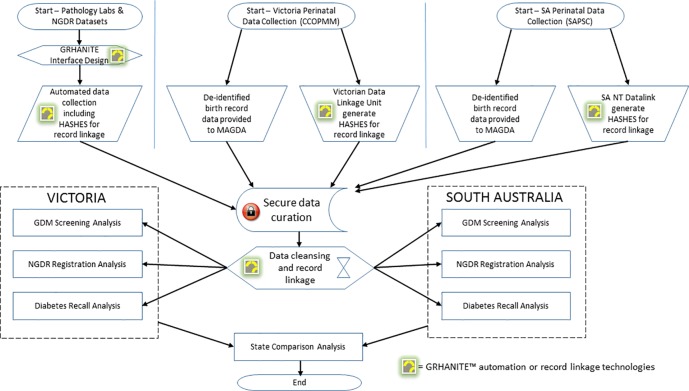
Study design (data acquisition, linkage and analysis). Note: ‘HASHES for record linkage’ refer to strings of letters and numbers generated by a one-way mathematical algorithm—that is, a function which it is infeasible to reverse or invert. Such algorithms are known as cryptographic ‘hash’ functions. Such hashes are generated whilst the data is still held by the original custodian of the data. When data is exported, person identifiers are left behind leaving only the non-reversible ‘hashes’. These are subsequently used to undertake privacy-preserving record linkage.

This study was part of a larger project, Mothers after Gestational Diabetes in Australia (MAGDA), which took a system approach to prevention of progression to type 2 diabetes among women previously diagnosed with GDM [4]. The study was approved by the relevant ethics committees; SA Health Human Research Ethics Committee HREC/14/SAH/93 and Department of Health, Victoria HREC 14/12. All data including NGDR, State perinatal, and pathology laboratory data was fully anonymised at-source with researchers having access to fully anonymised data only. Given the anonymous nature of the data, all ethics committees waived any requirement for patient informed consent. The study design is described below (Fig 1).

### Data sources and availability

Laboratory data came from the Australian states of Victoria (population 5,574,500) and South Australia [5] (population 1,645,000), which combined make up approximately 32% of the Australian population (2011 census data, Australian Bureau of Statistics, 2012). Data were supplied by four providers (SA Pathology, Healthscope, Melbourne Pathology, and Dorevitch Pathology). One provider (SA Pathology) processes samples from public hospitals and community samples; the other three primarily process private hospitals and community samples. These laboratories are the predominant pathology providers in the two states studied and for our study population (see results later). Diabetes-related test results from these laboratories during the pre and post-natal time period were found for 69% of our Victorian cohort and 82% of our South Australian cohort.

Birth records came from the Consultative Council on Obstetric and Paediatric Mortality and Morbidity (CCOPMM) in Victoria and South Australian Perinatal Statistics Collection (SAPSC). Reporting by hospitals is mandatory. Data were available in South Australia from 2008 to 2013 and in Victoria from 2009 to 2013. We accounted for birth plurality to ensure the total number of pregnancies were being considered rather than births.

GDM and type 2 diabetes register data were from the NDSS database, which has operated NGDR as a subset since July 2011. GDM and type 2 diabetes diagnostic information was available from 2008 to December 2015.

### Data acquisition and linkage

Legislation specifically prohibits release of person identifiers from CCOPMM and SAPSC. Privacy considerations for pathology laboratories were also a barrier to extracting person-identifiable data. To overcome this, we utilised a privacy-protecting data acquisition and linkage system (GRHANITE) [6]. GRHANITE creates probabilistic, hashed data linkage key signatures whilst data are still held within the bounds of the data custodian’s organisation. These signatures are based on surname, forename, date of birth, postcode, and 5 digits of the medicare ID when available. To comply with legislation the full medicare ID is never used. The algorithm for GRHANITE’s key generator was designed and tested alongside traditional person-identifiable data linkage mechanisms to validate that similar sensitivity and specificity data linkage profiles were achieved [7]. GRHANITE hashed signatures cannot be reversed, guaranteeing anonymity when extracted. The GRHANITE automated periodic data extraction and secure, encrypted data transmission from each data custodian organisation to the MAGDA Study GRHANITE databank were curated by The University of Melbourne.

The record linkage system gives no mechanism to manually validate linkage sensitivity and specificity because of its privacy-protecting nature, although these can be estimated. All mothers who are registered on NGDR should have a corresponding perinatal birth record with a GDM diagnosis. The days between NGDR registration and birth in the linked records were examined with an alignment between NGDR registration and birth expected. This relationship was examined for all registrations in Victoria and South Australia for 2012 and 2013.

### Study population

The overarching study population and record linkage included all records from the state birth records (CCOPMM and SAPSC), the NGDR and pathology laboratory data between 2008 and 2013 inclusive. Sub-populations were utilised with different start and stop dates for different parts of the analysis–for example data was analysed before and after the introduction of the NGDR to assess differences in laboratory testing after birth, and the core sub-population analysed activity from six months after the establishment of the NGDR until 2013. In some analyses, the use of first pregnancy only was applied to ensure mothers could not have had a previous GDM diagnosis and could not have participated in any NDSS datasets. These linked data contained data linkage keys, information on number of previous pregnancies, baby date of birth, maternal age at birth, GDM diagnosis (if present), pregnancy plurality, ethnicity, and geographical location (Local Government Area). The NGDR provided data on date of registration from register inception on 1 July 2011 until 31 December 2014. The NGDR has fixed dates after registration when bulk mail-outs occur so the study dataset itself did not contain actual dates of mail-out but the times when mail-outs to mothers and GP’s were undertaken for each mother was known. Data prior to commencement of the NGDR was available from 2008, although a GDM diagnosis was overwritten if a mother converted to type 2 diabetes before NGDR inception in July 2011. The NGDR data also included data linkage keys, expected baby date of birth, ethnicity, geographical location (Statistical Areas Level 2), mother’s age at registration, and socio-economic status (Index of Relative Socio-Economic Advantage and Disadvantage, 2011 Australian Census) [8]. Pathology data aimed to capture the majority of diabetes-related testing in the female population in Victoria and South Australia but logistical constraints meant it was not possible to collect data from all pathology providers. All blood glucose (BGLU), glycated haemoglobin (HbA1c), oral glucose tolerance test (OGTT), and glucose challenge test (GCT) data for women from 2008–2014 were extracted from participating providers. We collected data linkage keys, date of test (collection), type of test, test result, age at test, and geographical location (Local Government Area). For the main study analysis examining NGDR recall performance metrics, we concentrated on birth records from 2012 to 2013 and their corresponding registrations. This approach provided two years of registration data with a minimum of one year of follow-up laboratory testing data. We also examined rates of GDM screening and registration likely to have been triggered by NGDR.

### Defining screening, registration and recall

Pathology data (BGLU, HbA1c, OGTT, and GCT) were linked to the CCOPMM and SAPSC databases to estimate screening rates. GDM screening was recorded for a woman if a blood test result during the normal gestation period for a baby delivered in 2012 or 2013 was linked to a birth record, with the limitation that any blood test for glycaemia during pregnancy was treated as a possible GDM screen. The same linkage was used to estimate pathology coverage rates with the limitation that women who changed pathology provider to one not supplying us with data would not be accounted for. Because missing data affects those who have GDM and those without, mothers who use alternate laboratories do not affect the accuracy for assessment of GDM incidence. This assumes missing data was missing at random and we do not believe there is a specific reason that women would actively choose alternative laboratories–most will attend the laboratory specified by their clinician.The GDM screening variable was cross-tabulated with the CCOPMM and SAPSC database variable defining a pregnancy as GDM-affected. The rationale for this was based upon the assumption that all women should be screened. There should be no difference in the proportion of women screened, whether GDM was diagnosed or not.

The GDM register did not record the date of delivery–only the date of registration of GDM. The pattern of GDM register registration relative to the date of delivery was determined after record linkage and revealed a peak in registration at 68 days (9.7 weeks) prior to birth, aligned to 1–2 weeks after the near universal, but voluntary, GDM screening test carried out at 26–28 weeks. To ensure the temporal shift between registration and delivery was accounted for in comparative statistics, we included GDM registrations from the last 68 days of the 2011 calendar year in the numerator. Specifically, the 68 day temporal adjustment allowed a population comparison between the GDM register records and dates of delivery as recorded on the CCOPMM and SAPSC databases during 2012 and 2013. Because 68 days represents the peak alignment between the GDM register registrations and dates of delivery there will be some births missed in the population comparison due the vagaries of early births. The alignment is the closest that can be done to effect a comparison across the whole GDM register population and the whole population perinatal birth records.

**Fig 2:**
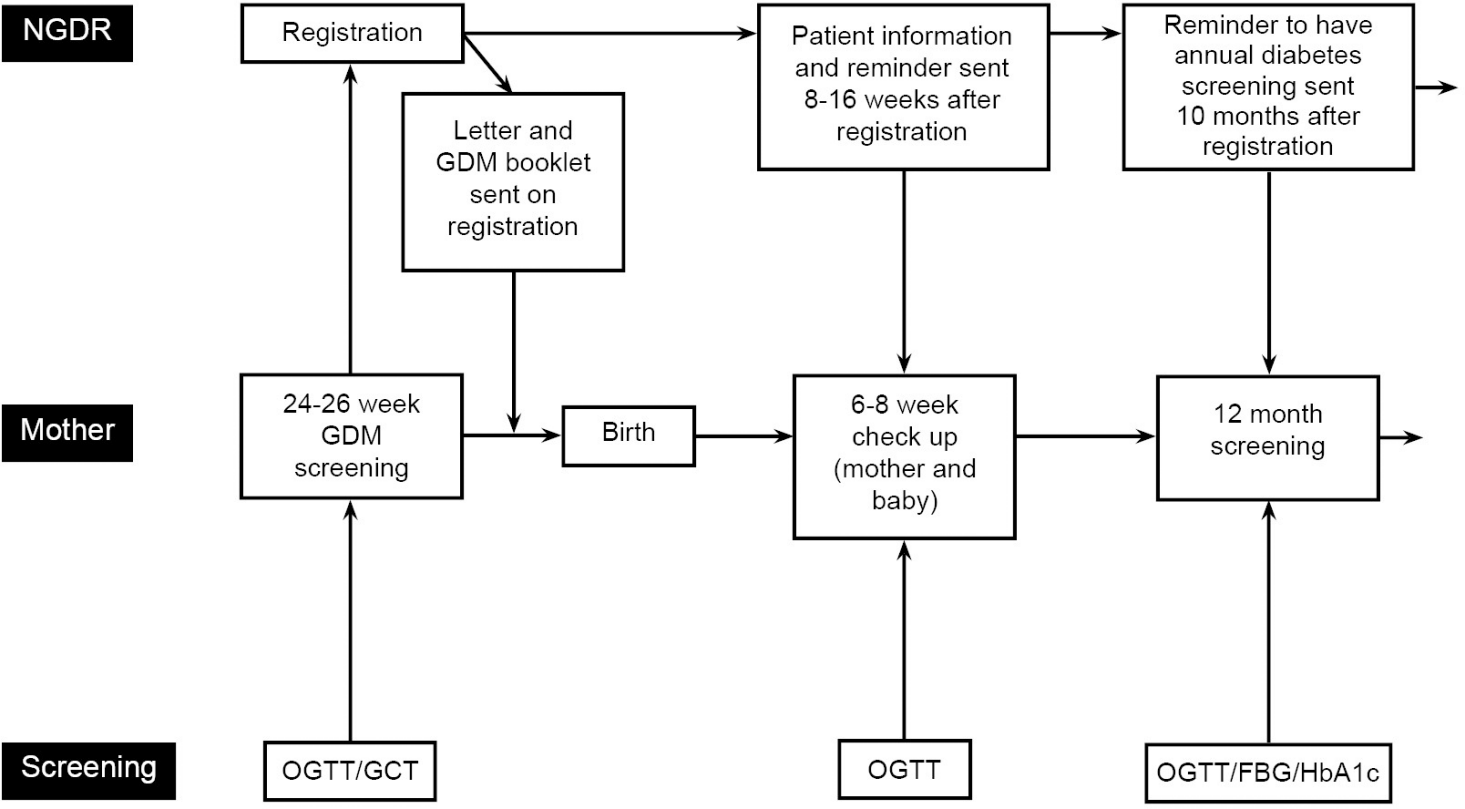
Timeline of GDM test and type 2 diabetes follow-up in relation to the NGDR NGDR—national gestational diabetes register.

Estimating the effectiveness of the recall was done by firstly defining the recall population. Only women with GDM are registered with NGDR. The woman’s date of registration was used in this analysis and we allowed up to 42 weeks gestation. NGDR recall was defined as a woman who should have received a reminder letter from the NGDR and then had a blood test at a time consistent with mail-out (i.e. women tested before mail-out were not classified as recalled by the NGDR (Fig 2)). Operational timelines for mail-outs were sourced directly from the NGDR. NGDR reminder letters are mailed out 8–16 weeks after registration (Fig 2). An analysis of follow-up diabetes screening was done by comparing the pattern of diabetes screening occurring prior to the NGDR’s establishment to further elucidate impact of the mail-out. We also examined patterns of diabetes screening during the first 12 months postpartum to capture any activity consistent with the NGDR annual diabetes screening reminder mail-out ten months after registration.

Data management, analyses, and visualisation were done in Microsoft SQL Server, Microsoft Excel 2013, and Stata 14 [9,10,11].

## Results

### Record linkage

A total of 12,235,355 data records and 6,714,844 data linkage keys contributed to the 2012 to 2013 analysis. Victoria had higher record numbers than South Australia, broadly reflecting the difference in each State’s population. Table 1 illustrates the raw record numbers prior to cleaning and data linkage with the OGTT, GCT, HBA1c, and BGLU columns being the total number of tests available from each laboratory. Of 13,316 records found on the NGDR for 2012–2013 (SA and Vic combined), after cleaning and record linkage12,214 mothers were recorded with GDM on the NGDR. Of these, 10,599 had corresponding birth records on state perinatal birth record systems (indicative linkage sensitivity 86.7%).

**Table 1:** Raw data availability in Victoria and South Australia (2012 and 2013). (Includes pathology data prior and subsequent to birth. HbA1c almost all used at one year follow-up).

**Victoria**							
**Laboratory**	**OGTT**	**GCT**	**HBA1c**	**BGLU**	**NDSS GDM Register**	**South Australia Birth Register (SAPSC)**	**Victoria Birth Register (CCOPMM)**
Laboratory A	35,284	10,262	150,483	594,620			
Laboratory B	27,817	11,538	144,564	590,330			
Laboratory C	51	146	905	10,131			
Laboratory D	52,319	15,626	80,442	484,385			
Total	115,471	37,572	376,394	1,679,466	10,819	332	150,031
**South Australia**							
**Laboratory**	**OGTT**	**GCT**	**HBA1c**	**BGLU**	**NDSS GDM**	**SAPSC**	**CCOPMM**
Laboratory A	9,177	5,055	49,394	95,649			
Laboratory B	5	4	0	120			
Laboratory C	19,333	30,942	104,966	1,312,377			
Total	28,515	36,001	154,360	1,408,146	2,497	40,599	5,973

**Fig 3:**
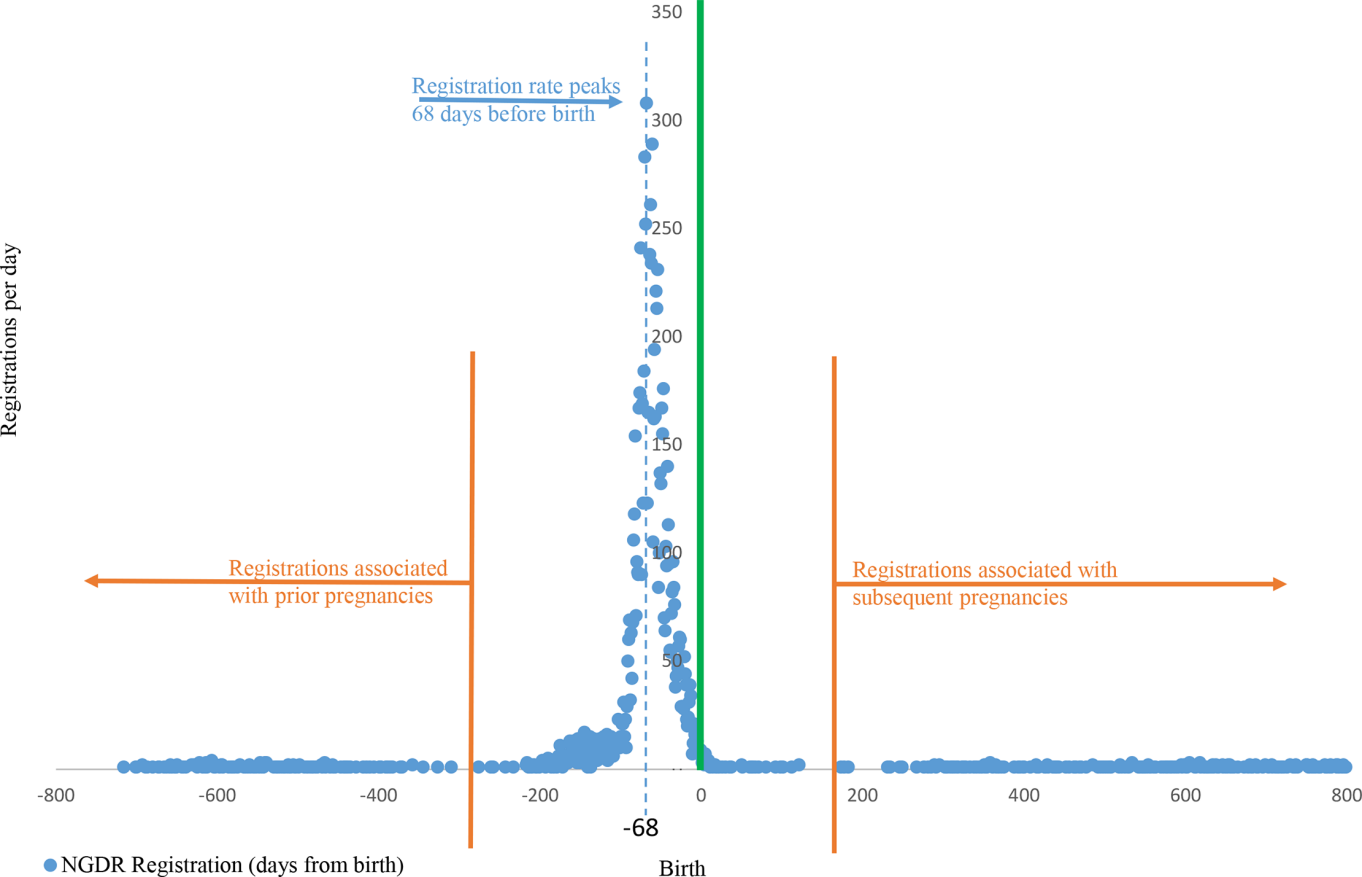
NGDR registration in days prior to and after baby birth for 2012 and 2013 NGDR—national gestational diabetes register.

NGDR registration peaked 68 days before the baby birth date (Fig 3) and inspection of the distribution revealed examples of linkages associated with pregnancies outside the study period of 2012–2013 (i.e. registrations related to prior or subsequent pregnancies).

### GDM screening

OGTT and GCT laboratory test data from 2009–2014 was linked to the state perinatal data collections for mothers who had a GDM diagnosis in the years 2010–2013. Table 2 details the number of women diagnosed with GDM 2010–2013 and gives the number of these women that we had prenatal GDM diagnostic laboratory tests available for. The table also illustrates the number of women who have a GDM screening test record available aligned to a routinely planned postnatal checkup at six weeks. Using this data, we aimed to estimate the percentage of mothers missing GDM screening in 2013:

Victoria:

- For mothers with no GDM diagnosis, screening data was available for 50.0%
- For mothers with a GDM diagnosis, screening data was available for 55.1%

South Australia:

- For mothers with no GDM diagnosis, screening data was available for 68.6%
- For mothers with a GDM diagnosis, screening data was available for 82.8%

**Table 2:** GDM screening and follow-up rates.

	**Victoria (Mothers with GDM diagnosis)**				
**Year**	**Women**	Screen available*	**%**	6 week follow-up test**	**%**
2010	3864	1434	37.1	710	49.5
2011	4212	1549	36.8	872	56.3
2012	5413	2672	49.4	1560	58.4
2013	6045	3330	55.1	1892	56.8
	**South Australia (Mothers with GDM diagnosis)**				
**Year**	**Women**	Screen available*	**%**	6 week follow-up test**	**%**
2010	1091	832	76.3	358	43.0
2011	1393	1112	79.8	470	42.3
2012	1468	1205	82.1	525	43.6
2013	1636	1355	82.8	599	44.2

* population where a prenatal GDM screening record (OGTT / GCT) was available

** population of those with an initial GDM diagnostic record where a further GDM screening record was available at the time of a 6-week postnatal checkup

In both South Australia and Victoria we observed that those with a positive diagnosis had a higher proportion of mothers tested for GDM than those without (55.1%–50.0% = 5.1% VIC, 82.8%–68.7% = 14.2% SA). Given that all mothers with a GDM diagnosis must have been screened, the lower rate of GDM in those without available test data may be explained by a population of mothers who were not screened.

From the above, in Victoria, 5.1% of patients who were not diagnosed with GDM (35,160 patients not diagnosed) must have missed GDM screening. We estimate this to be 1793 patients (5.1% of 35,160) out of a total population of 76,663 births, meaning approximately 2.3% of mothers missing GDM screening.

In SA, 14.2% of patients who were not diagnosed with GDM (5,746 patients not diagnosed) must have missed GDM screening. We estimate this to be 816 patients (14.2% of 5,746) out of a total population of 19,909 births, meaning approximately 4.1% of mothers missing GDM screening.

### NGDR registration

**Fig 4:**
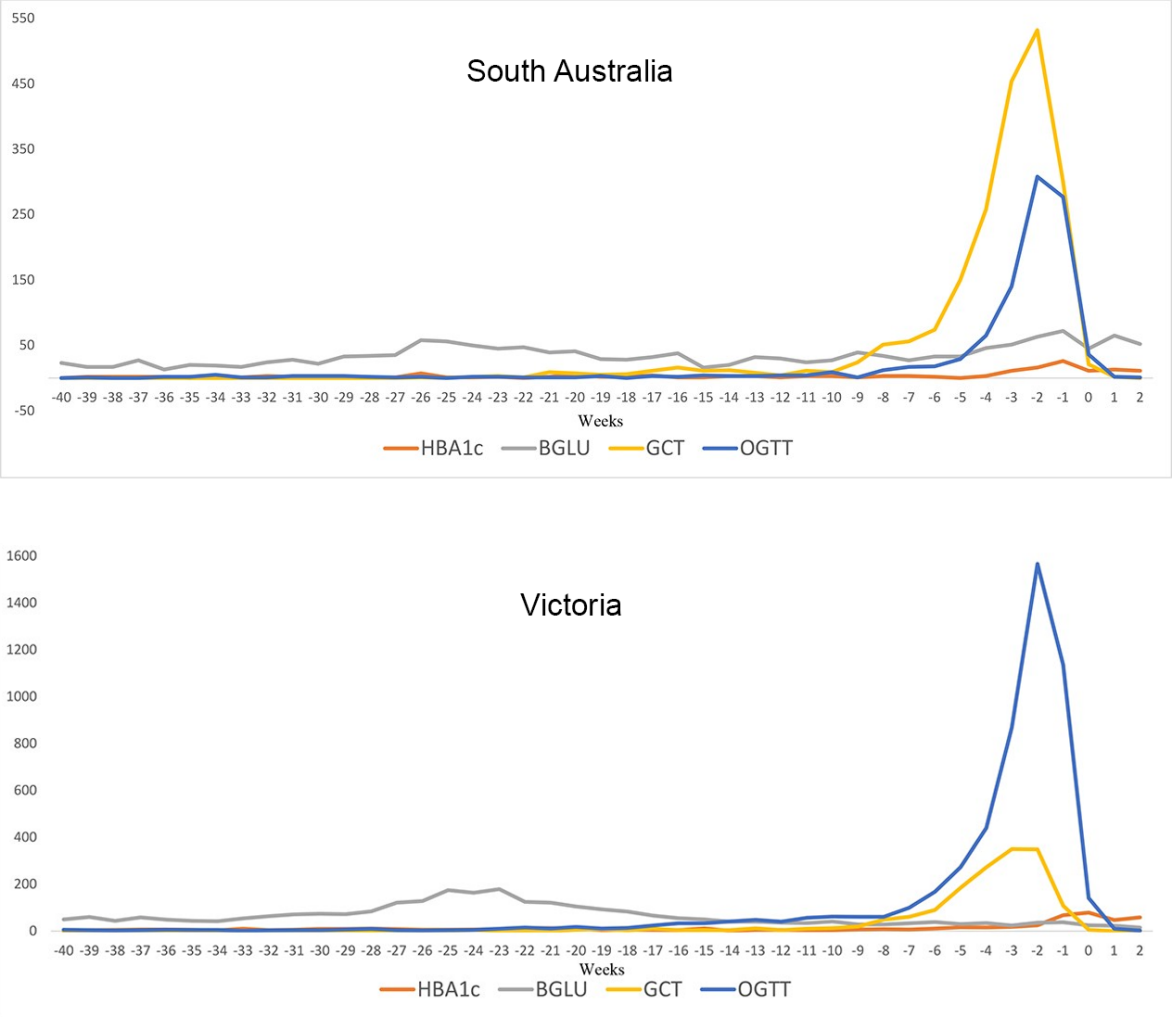
Pathology testing for 2012–2013 NGDR registered women in the weeks prior to GDM registration (at time 0).

The distribution of OGTTs and GCTs showed peaks consistent with screening for GDM using these tests prior to registration (Fig 4). Patterns in BGLU, including fasting glucose, and HbA1c were less obvious (Fig 4).

In Victoria from 2012 to 2013 GDM diagnosis rates increased from 7.0% to 7.9% whilst GDM register registration rates remained stable around 87%-88% (Table 3). In South Australia, GDM diagnosis rates rose from 7.2% to 8.2% whilst GDM register registration rates dropped from 78% to 70% (Table 3).

**Table 3:** GDM and NGDR registration rates 2012–2013.

	Victoria	Victoria	South Australia	South Australia
	2012	2013	2012	2013
Total Pregnancies	76,726	76,663	20,328	19,909
Pregnancies with a GDM diagnosis on birth record	5,413	6,045	1,468	1,636
GDM diagnosis rate	**7.0%**	**7**.9**%**	**7**.**2%**	**8.2%**
Pregnancies with NGDR registration	4,701	5,262	1,147	1,146
Registration rate	**88%**	**87%**	**78%**	**70%**

### Follow-up testing for risk of diabetes

**Fig 5:**
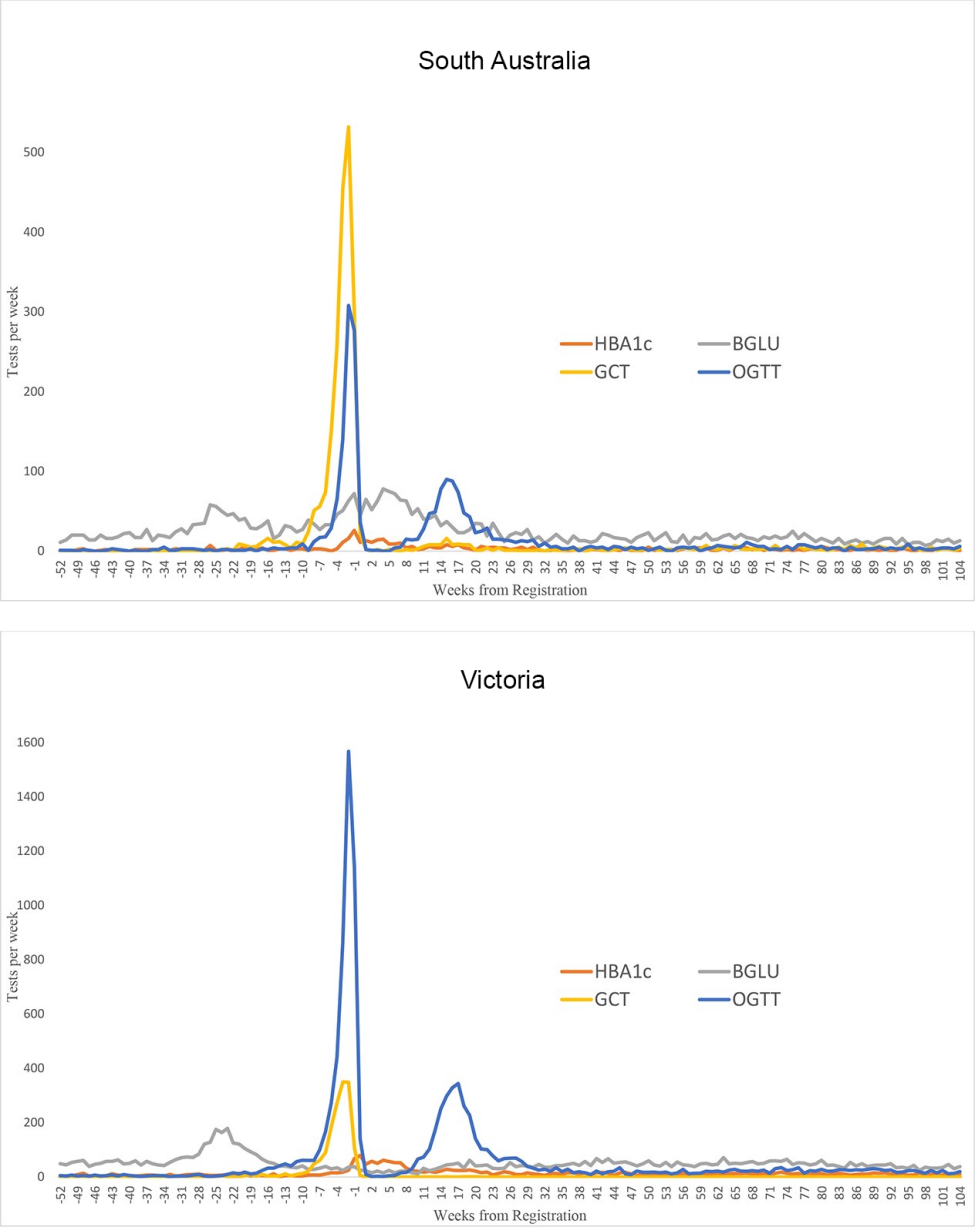
Laboratory testing in Victoria and South Australia relative to the date of GDM registration; 1 year before registration -> 2 years after registration.

**Fig 6:**
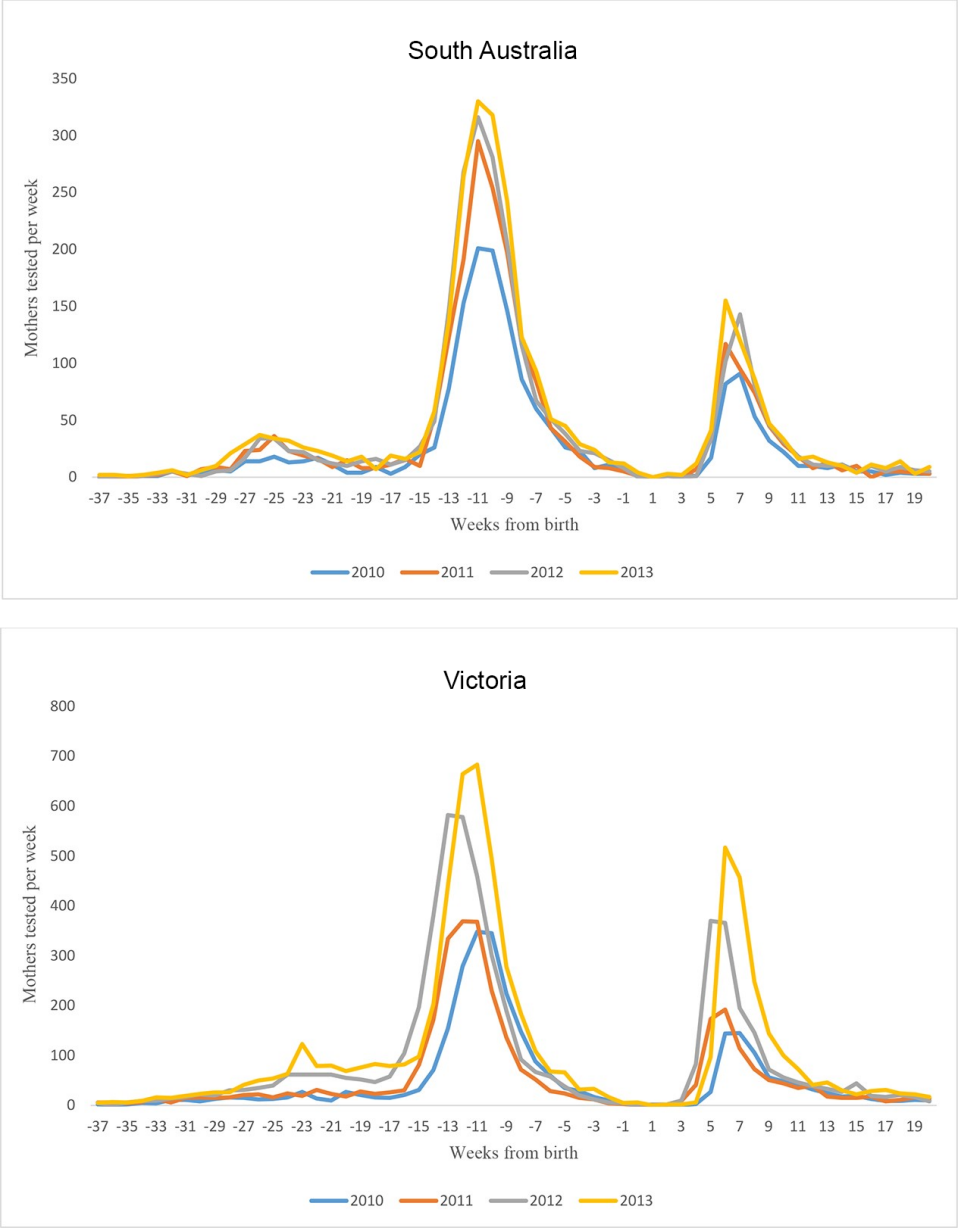
Screening and recall 2010–2013, Victoria and SA.

OGTT testing in the recalled population (see Methods) was most pronounced between eight and 30 weeks after registration (Fig 5). This period overlaps with the timing of NGDR reminder mail-out. The peak OGTT activity occurred almost exactly at six weeks postnatally. This can be seen in Fig 6 (almost all GDM screening activity is OGTT, 11% are GCT in South Australia). The six week postnatal activity suggests most follow-up occurs independently of mail-out (before women receive the letter) and corresponds to clinical best practice. We were unable to attribute testing activity after this point in time to the mail-out or women being followed up as part of usual care. We assume that if mail-out was having an impact it would prompt women who did not attend their six week test to have a test sometime after which would be picked up as another sharp spike in testing activity corresponding with the monthly mail-out from the NGDR.

To further assess potential influence of mail-out, patterns of six week postpartum follow-up testing were graphed for calendar years 2010–2013 (Fig 6). If the mail out, rather than a regular planned appointment, was influential in obtaining appointments for postnatal review a clear alignment of testing activity should have been evident in years 2012 and 2013 in response. This was not seen. Negligible activity at around 52 and 104 weeks postpartum (see Fig 5) suggests the mail-out is of little benefit for annual follow-up.

Absolute screening and recall rates cannot be determined due to incomplete pathology data coverage. Nonetheless, based on the number of diagnostic test results we have for mothers diagnosed with GDM in perinatal data collections (who must therefore have had at least one test undertaken), we were able to estimate that laboratory data available to us represented 69% and 82% of all GDM testing for Victoria and South Australia respectively. The South Australia state-specific diabetes register (prior to July 2011) demonstrated improved patterns of screening and stable levels of recall from 2010 to 2013 (Table 2). In comparison, Victoria exhibited a greater relative increase in both screening and recall activity over the same period (Table 2), noting this jurisdiction started from a lower base for screening and hence had more scope for improvement.

## Conclusions

The GRHANITE privacy-preserving linkage technologies enabled us to achieve the first successful linkage between different data sources, allowing an evaluation of the NGDR’s performance. Across 12.2 million clinical records, GRHANITE linkage identified the 26–28 week OGTT or GCT and six week postnatal OGTT results, but found no evidence of annual follow-up testing, prompted by reminder letters or otherwise, as demonstrated by a lack of diabetes-related blood testing activity at one year and beyond.

NGDR registration rates were higher in Victoria, perhaps related to a campaign to remind diabetes educators to register women. Although there was an increase in registration rates in 2013 in Victoria, the rate of new diagnoses of GDM in the birth registries outstripped the rate of new entries in the NGDR. With GDM diagnostic rates increasing, NGDR registrations should be increasing also. These were demonstrated to be static or falling. This finding shows the importance of periodically reminding diabetes educators about the register. Note that data acquired by this method could also be used to identify providers with poor registration rates.

Our estimated rates for GDM screening at 26–28 weeks are high at 97.7% in Victoria and 96% in South Australia (2012–2013).

To the best of our knowledge, NGDR is the world’s first national GDM registry. Evaluation of its performance is useful for others considering setting up similar systems. The sharp peak in type 2 diabetes diagnostic testing at six weeks is evidence of systematic six week appointments being made at time of birth. Reminder letters sent by the register to mothers and family physicians for screening follow-up are forecast to arrive after this appointment (according to NGDR timelines). There is no evidence that subsequent letters promoted further follow-up around 12 or 24 months.

Uptake of annual follow-up screening for type 2 diabetes was unsatisfactory (Fig 5) and lower than for other screening programs for women, such as breast screening 54% [12] Pap smears 56% [13] or bowel screening 40% [14]. One explanation for this low uptake of testing is that women who have had GDM do not consider themselves to be at high risk for development of type 2 diabetes [15]. Many people do not think diabetes is a serious health problem [16] which may be another reason for lack of annual follow-up. More concrete conditions, like cancer, show higher screening [17] because there is a pathology that people recognise and fear. Diabetes is abstract, asymptomatic, insidious, and needs lifelong commitment to prevention and management. Promoting a delayed benefit from lifestyle changes runs counter to the factors that we know to predict engagement in prevention activities.

It has been shown that if a woman has FPG <6.0 mmol/l at the time of discharge postpartum, she has a low likelihood of postnatal diabetes [18,19]. Follow-up screening could be more effectively targeted if instead of the six week OGTT, Fasting Plasma Glucose (FPG) was tested prior to discharge from hospitals. We know from women with type 1 diabetes that the insulin resistant effect of pregnancy declines rapidly, within minutes of the placenta being delivered. It can be argued that if FPG was normal in the immediate postnatal period it is likely to be so, six weeks later. It seems reasonable to do immediate postnatal fasting plasma glucose tests and only proceed to formal OGTT if FPG >6.0 mmol/l.

We did not account for the potential impact of the new International Association of Diabetes and Pregnancy (IADPSG) guidelines for diagnosing GDM as they were not introduced until after the 2012–2013 study period reported here [20,21]. Up to one third more women are forecast to be diagnosed with GDM than with previous criteria [19,22]. When the new criteria were introduced, no consideration was given to long-term follow-up of those women who are now diagnosed with GDM because of the change in criteria and who are likely to be at lower risk of developing type 2 diabetes. A further use of our data linkage will be for a longitudinal study with stratification of risk so that those at highest risk can be followed up more frequently and more intensive efforts made for recall.

Limitations of the study which arise from privacy protection include an inability to manually validate record linkage quality. Other mechanisms were employed to verify the linkage efficacy: almost 87% of all registrations had a corresponding birth record demonstrated by the record linkage. Erroneous linkage would show random associations between registration and birth which was not found. There were also very clear indicators of 26–28 week antenatal and six week postnatal GDM testing as well as close association between GDM diagnosis and subsequent NGDR registration (Fig 3). There is no such thing as perfect record linkage unless one-to-one unique identifier matching is possible. In large populations small errors (sensitivity or specificity) have a negligible impact on population trends. This study utilises population trends and differences in percentage between sub-populations in its analysis building on the confidence of the matching between registration and birth and pre and postnatal testing patterns.

Engaging women who have had GDM in follow-up programs is difficult [3,23]. Social marketing may be required to change the perception of prediabetes as a significant predictor of diabetes. Convenience, systems of care, and recall systems have been cited as important determinants of effective annual screening [24] and their provision in Family Practice does seem to improve women’s participation [4].

Routine booking of a six week postnatal appointment at the time of baby birth contrinuted to 43.0–58.4% of mothers obtaining type 2 diabetes follow-up testing at six weeks (Table 2). The register which sends out an information pack on first registration after the 28wk OGTT may act as a reminder to attend this pre-booked appointment with an increase in testing in Victoria of up-to 8.9% following the advent of the register (49.5% before, 58.4% 2012 –Table 2) (no change in South Australia where they had a register previously). From six weeks after the birth onwards there was no evidence of periodic register mail-outs having any effect whatsoever on encouraging mothers to seek further annual type 2 diabetes follow-up testing.

One use for the NGDR could be to populate Family Practice-based registers of women who have had GDM. Patients are more likely to act when the message comes from their family physician. Early evidence from Primary Care research suggests that managing recall and testing of women who have had GDM *at the local level is likely to be more effective than letters coming to these women* from a national source [4].
